# Lip Schwannoma—A Rare Presentation in a Pediatric Patient: Case Report and a Literature Review

**DOI:** 10.3390/diagnostics15141825

**Published:** 2025-07-20

**Authors:** Cinzia Casu, Mara Pinna, Andrea Butera, Carolina Maiorani, Girolamo Campisi, Clara Gerosa, Antonella Caiazzo, Andrea Scribante, Germano Orrù

**Affiliations:** 1 Oral Biotechnology Laboratory, Department of Surgical Sciences, University of Cagliari, 09124 Cagliari, Italy; cinzia.casu2@unica.it (C.C.); antonellacaiazzo2792@gmail.com (A.C.); germano.orru@unica.it (G.O.); 2 Unit of Dental Hygiene, Section of Dentistry, Department of Clinical, Surgical, Diagnostic and Pediatric Sciences, University of Pavia, 27100 Pavia, Italy; carolina.maiorani@unipv.it (C.M.); andrea.scribante@unipv.it (A.S.); 3 Freelancer in Cagliari, 09100 Cagliari, Italy; girolamo.campisi@gmail.com; 4 Unit of Pathology, Department of Medical Sciences and Public Health, University of Cagliari, 09124 Cagliari, Italy; clara.gerosa@unica.it; 5 Unit of Orthodontics and Pediatric Dentistry, Section of Dentistry, Department of Clinical, Surgical, Diagnostic and Pediatric Sciences, University of Pavia, 27100 Pavia, Italy

**Keywords:** oral schwannoma, lip schwannoma, oral mucocele, oral ultrasound evaluation

## Abstract

**Background/Objectives**: Schwannoma is a rare tumor, typical in young adults, originating from the myelin sheath that surrounds Schwann cells. It can occur in any part of the Peripheral Nervous System (PNS). It develops in the head and neck region in 25–48% of cases, and the eighth pair of cranial nerves (vestibulocochlear nerves) are the most hit (vestibular schwannoma). Oral cavity involvement is exceedingly rare, accounting for about 1–2% of all cases. The most affected oral site is the tongue, especially its anterior third, while localization on the lip is one of the least common sites for the development of this lesion. **Case Presentation**: A lower lip schwannoma on a 17-year-old boy, present for about 7 years, was documented. **Material and Methods**: PubMed and Google Scholar were used as research engines; English scientific works published in the last 20 years (2005–2024) regarding oral cavity involvement, using the keywords “Schwannoma”, “Oral Schwannoma”, “Pediatric Oral Schwannoma”, and “Schwannoma of the lip”, were considered. **Results**: In total, 805 and 16,890 items were found on PubMed and Google Scholar search engines, respectively. After title, abstract, full text evaluation, and elimination of duplicates, 26 articles were included in the review process. **Discussion**: Clinically, oral schwannoma presents as an asymptomatic hard–elastic fluctuating mass, often misdiagnosed on the lip as a traumatic or inflammatory lesion (e.g., mucocele). Biopsy is mandatory, and histological examination reveals positivity to the neuronal marker S-100. **Conclusions:** Complete excision also prevents recurrence. Malignant transformation is extremely rare.

## 1. Introduction

Schwannoma, also called Neurinoma or Neurilemmoma, is a rare and benign tumor that originates from the myelin sheath surrounding the nerves of the Peripheral Nervous System [1,2,3,4,5,6,7,8,9,10,11,12,13,14,15,16,17,18,19,20,21,22,23,24,25,26], due to an uncontrolled proliferation of Schwann cells which compose the myelin sheath, discovered at the beginning of 20th century by the neuropathologist Juan Verocay. It is characterized by slow growth and a firm–elastic consistency. It usually presents as a painless cyst-like lesion without signs of ulceration unless there is trauma [1,2,3,4,5,6,7,8,9,10,11,12,13,14,15,16,17,18,19,20], mimicking benign traumatic lesions, with which it leads to a differential diagnosis—i.e., mucocele, fibroma, lipoma, benign salivary gland tumors [1,2,4,6,17,21,23]—or other neurogenic tumors (neuroma, neurofibroma). It typically affects the head-and-neck region, hitting the eighth cranial nerve the most (giving origin to the so-called vestibular schwannoma); oral presentation is instead extremely rare. It is believed it has no gender preference [2,3,4,5,6,10,16,20,22,25], and it is more typical of young adults under 50 [1,2,4,5,16,20,21,23,25,26]. A diagnosis of certainty is only based on histology, obtained by biopsy, even if there is increasing evidence in the scientific literature that ultrasound is a tool with sensitivity and specificity almost comparable to histology in the treatment of oral exophytic lesions, with an accuracy of about 90% [22]. Once the nature of the lesion is ascertained, the treatment of choice is surgical excision [1,2,3,4,5,6,10,11,16,19,20,21,22,23,24,25,26]. Prognosis is good, with an exceptionally low tendency for malignant transformation [1,2,3,4,5,6,11,21,23,24,25,26].

## 2. Case Presentation

**Figure 1:**
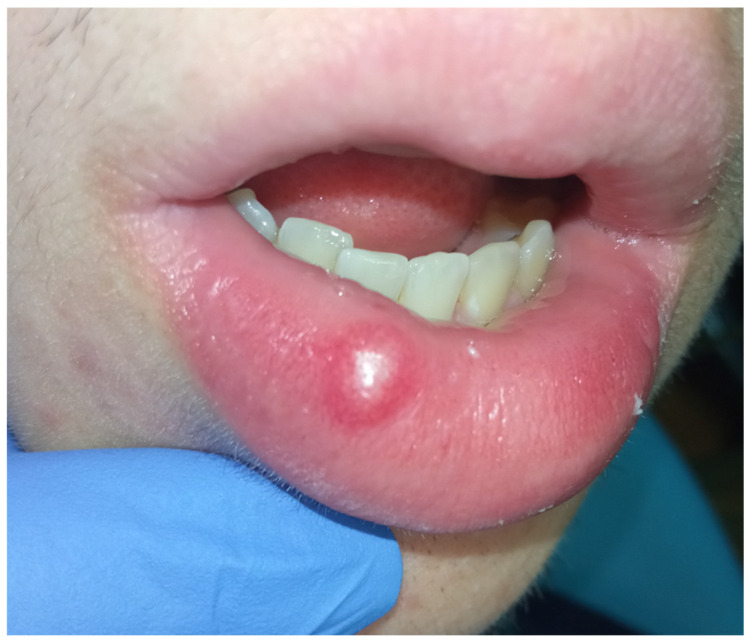
First clinical examination of the exophytic lesion.

We present a case of a 17-year-old boy who came to our attention showing an exophytic lesion localized on the right side of the lower lip which was present for about 7 years (Figure 1). His parents denied a pathological history of significant relevance. At the extraoral clinical examination, the lesion appeared monochromatic and floating. The boy did not refer to any kind of pain, but he and his parents reported that they saw it growing and increasing in volume over the years. He reported instead that the lesion is often accidentally traumatized, which always brought bleeding and a succeeding thickening of the lesion itself.

**Figure 2:**
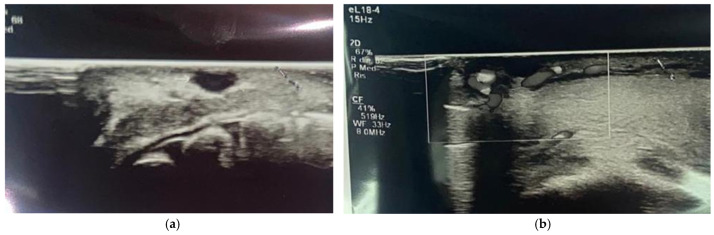
(**a**,**b**) Extracts from the labial ultrasound. Note the absence of vascularization or noble structures around the neoformation.

Due to the anamnesis and the clinical observation, we made a hypothetical diagnosis of a mucocele of the lower lip, a common lesion of traumatic nature, which was, moreover, agreed upon by other clinicians who visited the young boy. However, we decided to plan an excisional biopsy to determine the true nature of the lesion by a histological evaluation, an option that was never mentioned before our visit. First, we prescribed a labial echography with the task of identifying any kind of vascularization or noble structures such as terminal nerves in the perilesional area. As shown in Figure 2a,b, none of these were detected, so we proceeded with the excisional biopsy.

We performed it using a cold blade scalpel after a local perilesional injection of anesthetics (1.8 mL cartridge of mepivacaine + adrenaline 1:100.000). The incision showed a fuzzy, greyish lesion of about 1 cm in diameter, which was taken and placed immediately in a solution of 4% buffered formaldehyde for histological analysis. We put two silk stitches, and we set a control appointment for the next week to evaluate the surgical site and to eliminate the stitches, too. The histological analysis revealed a diagnosis of an oral schwannoma, which was a surprising outcome due to the extreme rarity of this kind of tumor in general and if located in the oral cavity.

**Figure 3:**
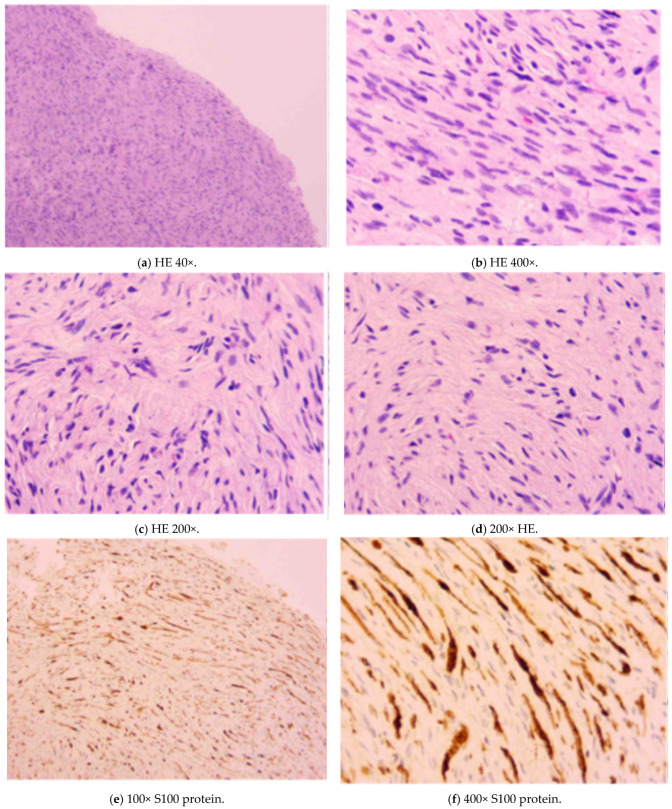
Histological analysis. (**a**) Microscopically, hypercellular areas (Antoni A) and hypocellular areas (Antoni B) were observed at low magnification. (**b**) This figure shows that the lesion was encapsulated and characterized by a proliferation of spindle cells, with no evidence of necrosis, mitotic activity, or significant nuclear atypia. (**c**) This figure shows the Antoni A areas, where spindle cells were arranged in palisading patterns, with the presence of Verocay bodies (acellular eosinophilic structures located between parallel rows of nuclei). (**d**) We can see that in the Antoni B areas the spindle cells were scattered within a loose fibrous stroma. (**e**,**f**) In these images, we can see with immunochemistry that the lesion showed diffuse positivity for S100 protein, supporting its neural origin. HE (Hematoxyline-Eosin) is the standard coloring used to analyze tissues.

The histological exam reported a lesion presenting “spindle nerve cells arranged in bundles, absence of necrosis, atypia and mitotic activity and positivity to the S100 protein spindle nerve cells arranged in bundles, absence of necrosis, atypia and mitotic activity and positivity to the S100 protein” (Figure 3).

At macroscopic examination, the lesion measured approximately 1 cm and was covered by intact skin with a parenchymatous consistency. On the cut section, the surface appeared whitish-yellow and slightly heterogeneous. Microscopically, hypercellular areas (Antoni A) and hypocellular areas (Antoni B) were observed at low magnification (Figure 3a). The lesion was encapsulated and characterized by a proliferation of spindle cells, with no evidence of necrosis, mitotic activity, or significant nuclear atypia (Figure 3b). In the Antoni A areas, spindle cells were arranged in palisading patterns, with the presence of Verocay bodies (acellular eosinophilic structures located between parallel rows of nuclei) (Figure 3c). In the Antoni B areas, spindle cells were scattered within a loose fibrous stroma (Figure 3d). At immunohistochemistry, the lesion showed diffuse positivity for S100 protein, supporting its neural origin (Figure 3e,f). The surgical resection margins were focally involved, which likely led to the subsequent recurrence of the lesion.

**Figure 4:**
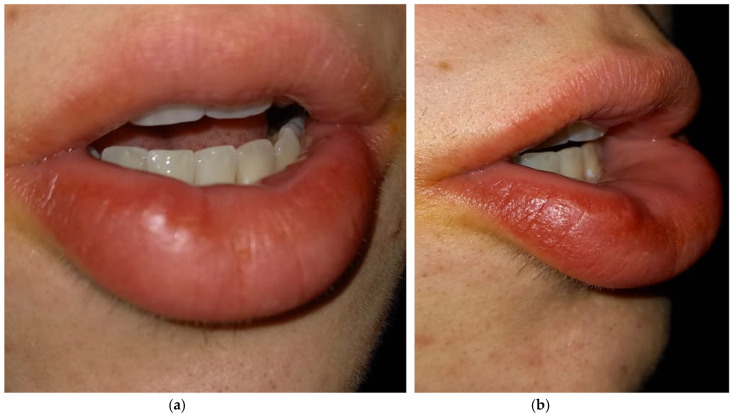
(**a**,**b**) One-month follow up: note the relapse of the lesion but in a minor dimension. (**a**) frontal view; (**b**) right view.

**Figure 5:**
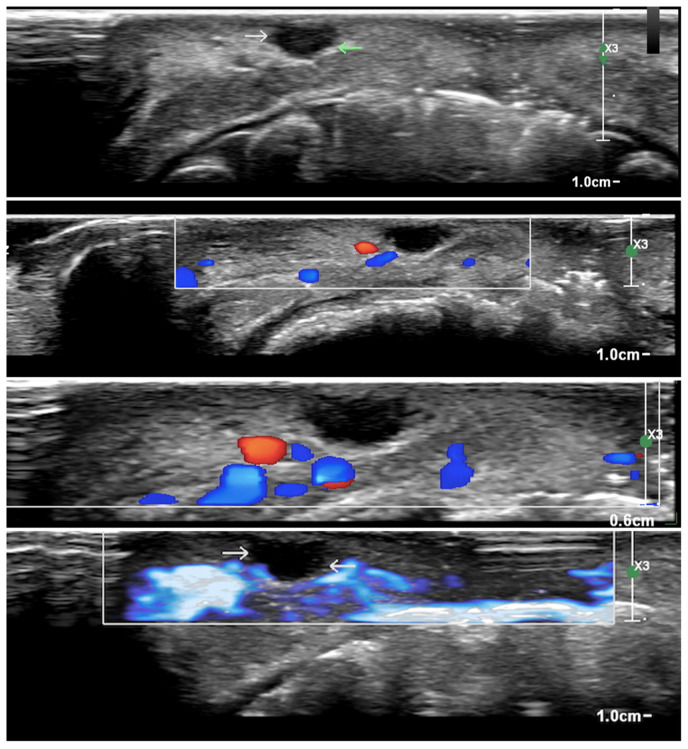
Extracts from the echocolor Doppler images. The ultrasound examination demonstrates the presence of a transonic, oval-shaped mass in the lower lip, with well-defined and sharp contours. No evidence of intralesional vascularization is observed. Blu areas refer to small venous vessels, red areas to small arterial vessels. In the four ultrasound images, a transonic mass with sharp and well-defined contours is clearly visible, lacking vascularization on color power Doppler interrogation. Peripheral vascularization is present, with a more significant vascular pole on the lateral right side. The role of ultrasound imaging in the study of soft tissues is well known. The examination uses ultrasound waves, physical elements that have no pathological effects on the patient or the operator. This method allows for the exploration of various regions of the human body. At the level of the lips and tongue, it is possible to examine any masses both in terms of tissue structure and, with the aid of color power Doppler, the vascularization of the lesions [27].

We set another control appointment a month later to establish if the lesion occurred again, due to its tendency to relapse. At the observation, we noticed a newly formed smaller lesion in the same area where we had operated (Figure 4a,b). So, we decided to prescribe a new echography—this time an echocolor Doppler ultrasound—in order to establish margins, the actual size of the lesion, and its growth (Figure 5). The exam showed the recurrence of the lesion, although extremely limited compared to the previous one. The decision we took this time was to not set a second excisional, and this decision was taken together with the sonographer and the boy’s general practitioner (whose specialization is in oncology), first because the benign kind of the lesion was already attested and secondarily because the incision could cause labial paresthesia given the true nature of the lesion. We instead set close follow-up appointments due to the tendency towards the growth and development of the schwannoma. The boy and his parents agreed. Moreover, we informed our patient that schwannoma shows a consistent association with the syndromic picture of Neurofibromatosis type 2 (NF2).

## 3. Materials and Methods of the Review

We performed a review of the literature using PubMed and Google Scholar as a research engine. We set some inclusion and exclusion criteria (Table 1) in order to fulfill our review. We set as inclusion criteria scientific work published in the last 20 years (2005–2024) as case reports, clinical trials, meta-analysis, reviews, and systematic reviews of the literature written in English. Exclusion criteria included articles written before 2005 and not in English language. The keywords used are “Schwannoma”, “Oral Schwannoma”, “Pediatric Oral Schwannoma”, and “Schwannoma of the lip”. After reading the abstract and full text, we included scientific articles in which the primary disease was localized in the head and neck region, with particular attention paid to the oral cavity.

## 4. Results

The research was performed using the PRISMA protocol for new systematic reviews. After screening 16,995 records, we excluded 16,969 irrelevant titles/abstracts, leaving 26 eligible articles for full-text reviews. The first selection allowed us to narrow our research on Google Scholar engine and eliminate duplicates (14 items discarded) and incoherent records by automation tool (15,334 records discarded); the second selection (*n* = 1647) of records occurred after reading titles and abstracts on both research engines, leaving 40 articles (1607 items discarded). The final selection occurred after full-text reading and brought us to discard 14 articles, because they did not involve the oral cavity. The final selection for our revision considered 26 articles.

**Figure 6:**
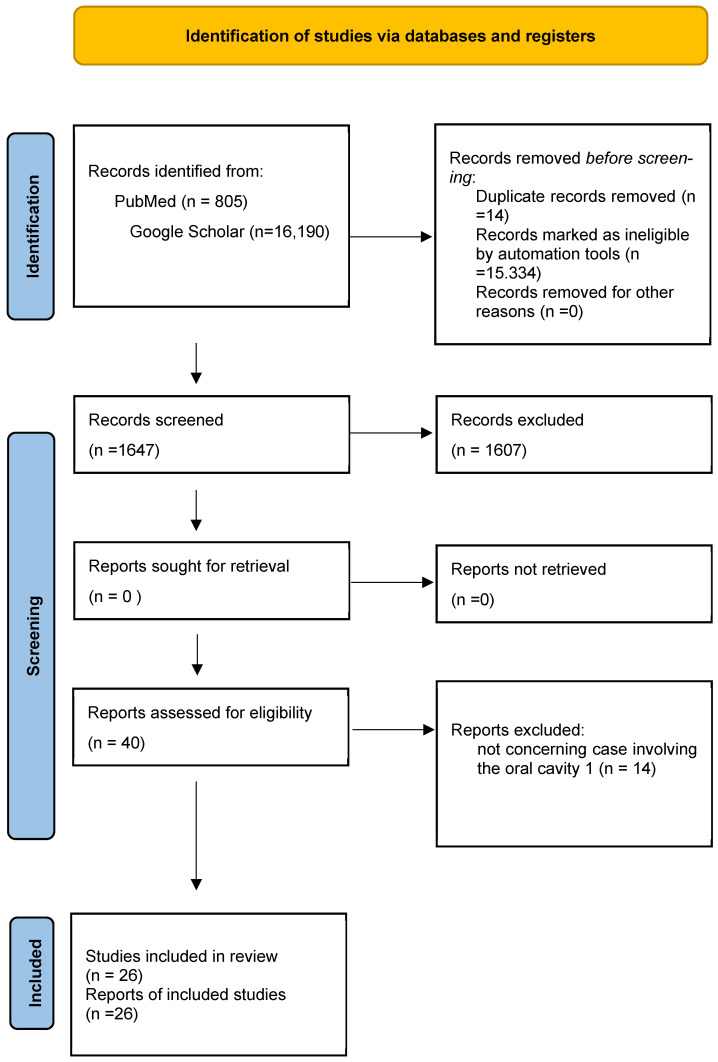
PRISMA flow chart showing the process of choosing articles which were finally included in the literature review.

The flow chart below (following the latest PRISMA guidelines) illustrates schematically the procedure used for the article research (Figure 6). Starting with PubMed, the first search brought 721 results for the “Schwannoma” keyword, 33 results for the “Oral Schwannoma” keywords, and 51 results for “Pediatric Schwannoma”. The second selection occurred after reading the abstract, and 17 articles for “Schwannoma”, 7 articles for “Oral Schwannoma”, and 4 articles for “Pediatric Schwannoma” were selected to be further analyzed. The third selection of articles led to the selection of 2 articles for the keyword “Pediatric Schwannoma”, 10 articles for the “Schwannoma” keyword, and 6 articles for the “Oral Schwannoma” keywords. The entire reading of the articles brought us to consider a total amount of 18 articles from the PubMed research engine.

Research using Google Scholar brought 9820 results for the “Pediatric Oral Schwannoma” keyword and 6370 results for “Schwannoma of the lip”. Given the large number of results unrelated to our research, we decided to narrow the focus exclusively to lesions of the oral cavity, thus using keywords such as “pediatric oral schwannoma-vestibular, -gastrointestinal, -spinal, -plexiform, -oropharyngeal, -palsy, -mucositis, -laryngeal, -cerebellar, -vagal, -parotid, -lymphangioma, -intraosseous, -dental, -infraorbital, -osseous, -hemangioma, -pelvic, -uterine, -nevi, -nasal”, a search that led to a primary identification of 414 articles. After reading the titles and abstracts, we selected four articles relevant to our research for the first keyword (Table 2). Also, for the second keyword, we narrowed the focus exclusively to labial lesions, thus entering the following into the search engine: “schwannoma of the lip-ancient, -cheek, -palate, -face, -tongue, -neurofibroma, -lingual -vestibular, -gingiva, -mandible, -rhomboid, -melanotic, -plexiform, -papule”, a search that led this time to 442 results. After reading the titles and abstracts, we selected 12 articles. The accurate reading of these articles brought us to almost discard six of them because the other six did not fit the inclusion and exclusion criteria; two articles with the keyword “Pediatric Oral Schwannoma” were selected because they were fully relevant to the search criteria. Therefore, the total number of articles considered in this review is 26.

## 5. Discussion

### 5.1. Generalitis

#### 5.1.1. Definition

Schwannoma is a rare and benign tumor that originates from the myelin sheath surrounding the nerves of the PNS (Peripheral Nervous System) [1,2,3,4,5,6,7,8,9,10,11,12,13,14,15,16,17,18,19,20,21,22,23,24,25,26]. Although Masson in named this lesion in 1932, the aforementioned neuropathologist Juan Verocay histologically described this entity in 1910, discovering the presence of bands of eosinophilic amorphous substance between the nuclei of the cells involved [1,2,3,4,5,10,16,17,21]. This feature, named “Verocay bodies” after him, still characterizes all neuronally derived tumors.

#### 5.1.2. Epidemiology

In the literature, 25–48% of all schwannomas reported developed in the head-and-neck region [1,2,3,4,5,6,7,10,11,16,17,19,20,21,23,24,25,26], and oral presentations are considered extremely rare, accounting for about 1–2% of all cases [1,2,3,4,5,6,11,16,17,19,20,21,25]. Most of the authors found no gender preference [2,3,4,5,6,10,16,20,23,25,26], although Salehinejad et al. [5] reported a slight predilection for the female sex considering schwannomas of the oral cavity.

This type of tumor can develop at any age, although it is more typical of young adults, with the highest number of diagnoses occurring in the decade between 20 and 50 years old [1,2,4,5,16,20,21,22,23,24,25,26]; it is very rare in pediatric age [20,23,24], and the frequency with which it is found in the oral cavity is higher in adults than in children [1].

#### 5.1.3. Etiology

The etiology is still unknown [4,5,10,16], but there is a fascinating theory about a possible link between the development of schwannoma and a traumatic injury: it is called “Failure of nerve regeneration” and it consists of a failure of Schwann cells in regenerating themselves after a single nerve injury [12] Basically, when injured, a Schwann cell lacks the capacity to re-differentiate into myelinated cells and undergoes uncontrolled proliferation [11,12]. This phenomenon is encouraged by the mutation of both copies of the NF2 gene, which controls proliferating pathways.

#### 5.1.4. Clinical Features and Localization

Schwannomas present as a slowly growing mass [1,2,3,4,5,6,10,16,17,19,20,21,22,23] and have cystic-like exophytic lesion presentation and a firm–elastic consistency. They are usually painless, neither spontaneously nor upon palpation, and they do not present ulceration unless there is trauma [1,2,3,4,5,6,7,8,9,10,11,12,13,14,15,16,17,18,19].

There are central or intraosseous schwannomas, which can cause pain, paresthesia, and bone expansion [1,2,5,10,11,17], or the peripheral variant [1,11,17]. In the oral cavity, the intraosseous schwannoma is predominantly found in the mandible [10], which is also the most affected site by this variant in the head and neck region, while the peripheral schwannoma is found in the soft tissues. Schwannoma in the oral mucosa often presents as a solid mass of variable measure, mimicking benign traumatic lesions such as mucocele, fibroma, lipoma, benign salivary gland tumors [1,2,4,6,17,21,23], or, on a more advanced level, other neurogenic tumors (neuroma, neurofibroma) [1,10].

The oral site most affected is the tongue, particularly its anterior portion [1,2,3,4,5,6,10,16,17,21,22,24,25]; here, if large, the tumor may cause discomfort and sometimes dysphagia and dyspnea [10]. There is agreement in the literature that other sites in the oral cavity are less affected, with the palate, vestibular mucosa, and floor of the mouth being represented in this order [1,3,4,5,6,17,24]. Lip presentation is extremely rare [2,23,24,25]: Maulane et al. in 2024 reported only 12 cases of schwannoma of the lip, of which 4 were found in pediatric patients (<20 years old) [21]. Seven cases of upper lip schwannoma were recorded by Bayindir et al. in their review in 2013 [22], supporting the rarity of this lesion.

There are two types of oral soft tissue presentation. When presenting as a firm–elastic floating mass, it is thought to be the encapsulated form, being circumscribed by a consistent layer of fibrous connective tissue with a smooth surface [1,2,3,4,6,7,10,11,15,16,17,19,20,22,23], which is the most common type of presentation, and it is also defined as a submucosal nodule [5,17]; the second form of presentation is the non-encapsulated one or pedicular form, commonly found in the basal layer of the mucous membrane [1,5,11,17].

Given that the tumor develops from Schwann cells (which form the myelin sheath of the axons of the PNS) [1,2,3,4,5,6,7,10,11,12,15,16,21,22], it can originate from all cranial nerves except the first two, the olfactory (I) and optic (II) nerves, which are part of the CNS (Central Nervous System) [1,10,22], while the most affected is the vestibulocochlear nerve (VIII) [4,12]. It can involve both motor and sensory nerves [1], with a preference for the latter [11], as well as from sympathetic fibers [1,2,5,10] and autonomic fibers [10]. The other cranial nerves most affected, according to Baderca et al. in 2008, are the glossopharyngeal (IX), vagus (X), accessory (XI), and hypoglossal (XII) nerves [10]. Helbing et al. 2020, in their article, also listed the VIII and XII cranial nerves as the most frequently affected but additionally propose the trigeminal nerve (V) [12].

Most extracranial schwannomas typically originate from the vagus nerve [5].

#### 5.1.5. Diagnosis

Clinical diagnosis is difficult due to the nonspecific characteristics with which the lesion presents. As previously mentioned, it enters differential diagnosis with the main and most common benign lesions of both traumatic and inflammatory nature that can commonly occur in the head and neck region [1,2,4,6,17]. The clinical assessment should be accompanied by radiological investigations, where CT (Computed Tomography) and especially MRI (Magnetic Resonance Imaging) are the gold standard exams for evaluating the size of the lesion and the extent of its margins [2,3,5,20,22]. Bayindir et al. in 2013 reported that in “ultrasonographic imaging, homogenous and hypoechoic findings and posterior acoustic enhancement” can be found [22], so a typical X-ray pattern can be assumed. Some studies report that FNAC (Fine Needle Aspiration Cytology) is essential to establish whether the tumor is malignant or benign [3], but these data are controversial and in contrast with other studies [1]. The gold standard remains biopsy followed by histological analysis [1,2,3,4,20,21], which will indicate a tumor lesion with a strong positivity for the S-100 protein. Histology is also the only method to establish the differential diagnosis with neurofibroma, which shares the precursor cell, the Schwann cell, from which the tumor proliferation originates [10].

In differential diagnosis with this lesion, benign pathologies, such as fibromas, epulides, lipomas, gingival hyperplasia, giant cell granulomas, mucoceles [4,6,17,23], or sometimes even neoplastic conditions, such as salivary gland tumors [4,17], are considered. When considering other neuronally derived tumors [1], the primary differential diagnosis is with neurofibromas [1,4,10,11], which share the same precursor of neoplastic transformation, the Schwann cell.

#### 5.1.6. Histopathology

On an immunohistochemical level, schwannomas, like most neurogenic tumors, show strong positivity for the S-100 protein, an important marker of the CNS [1,2,4,5,6,10,11,16,21,23,26]; Martins et al., in their 2007 paper, also reported positivity for EMA (Epithelial Membrane Antigen), fibronectin, CD34, and collagen, especially when the tumor is found in its encapsulated form [2]. Baderca et al. in 2008 also reported positivity for monoclonal antibodies against vimentin and the GFAP (Glial Fibrillary Acidic Protein) [10]. Another molecule that, according to some studies, might be even more specific and sensitive for diagnosing this tumor is SOX-10 [11].

The histopathological pattern of schwannomas is typical and presents as a mix of areas with high cellularity and low cellularity, referred to as Antoni A and Antoni B, respectively [1,2,4,5,6,10,11,16,17,21,22,23,24,26], according to an architectural criterion. The hypercellular areas contain spindle-shaped cells with elongated nuclei, well organized in palisades [1,2,4,5,10,11,12,16,17,26] and accompanied by the presence of so-called Verocay bodies [1,2,4,5,10,12,16,17,21,24]. These areas represent the most found histological pattern [2,11]. The hypocellular areas, on the other hand, are characterized by a rearrangement of cells embedded in a matrix of loose fibrous tissue [1,2]. Some authors [6,12,17] believe that the transition from high cellular density to low density—from Antoni A pattern to Antoni B—is crucial for its malignant degeneration, thus defining real “transition zones” at the edges. This might explain why the Antoni A pattern is more commonly found and more associated with the benign nature of the tumor.

#### 5.1.7. Treatment

The treatment of choice of schwannoma is surgical excision [1,2,3,4,5,6,10,11,16,19,20,21,22,23,24,26] under local anesthesia if the tumor is in the oral cavity. In the encapsulated form, a single excision is possible [1,5], which also lowers the recurrence rate [5], generally estimated in the literature as incredibly low or even null [1,2,5,6,10,11,16,21,22,23,24,25,26]. The non-encapsulated form, on the other hand, is more complicated to remove, as it is not circumscribed, making it more prone to recurrence [1]. With the necessary differences regarding the site where the lesion is located, the greatest difficulty in removing it remains the preservation of the nerve from which it originates [1].

#### 5.1.8. Prognosis

Sitenga et al., in their 2017 systematic review, reported a recurrence rate of 5.3% [19]. There is full agreement in the literature that malignant transformation is quite rare [1,2,3,4,5,6,10,15,23,25,26]. Nassehi et al., in 2021 [3], reported a malignant transformation rate of 8–10% for schwannomas in the head and neck region, which is roughly consistent with the data from Salehinejad et al. [5], who reported a rate of 9–14%. However, both studies agree that malignant transformation is extremely rare in oral cavity schwannomas [3,5].

#### 5.1.9. Association with Systemic Diseases

Multiple vestibular schwannomas, particularly bilateral ones, are associated with Neurofibromatosis type 2 (NF2) [3,4,5,9,10,11,12,13,16,18]. Nives Pećina-Šlaus, in her article (2013), reported up to 4% of such lesions in NF2 patients [15].

Although the main purpose of this review is not to focus on the genetic characteristics of schwannomas, it is important to briefly mention the molecular mechanisms that lead to their onset. It is now unanimously accepted in the literature that the origin of schwannomas is associated with a mutation in a cytoskeletal protein called Merlin, Neurofibromin 2, or schwannomin [15], which belongs to the ERM (Ezrin–Radixin–Moesin) protein family [8,13,15,21] and is regulated by the tumor suppressor gene NF2. This gene is located on chromosome 22, more specifically at the 22q12.2 region [14,15,18], and its mutation is associated with the onset of Neurofibromatosis type 2 [8,9,12,13,14,15,18]. The Merlin protein—the product of this gene—participates in many regulatory functions and acts in different cellular compartments [12,13,14,18]. At the membrane level, for example, it regulates cell adhesion through the activation of receptors for growth factors (GFs) and for intracellular signaling induced by contact in the extracellular matrix (ECM) [12]; at the cytoplasmic level, it inhibits both Ras and Rac G proteins, as well as several kinases [12,13,14,18]; at the nuclear level, it suppresses the activity of a particular ubiquitin ligase (CRL4DCAF1), which regulates the Hippo pathway, one of the main systems controlling cell proliferation and apoptosis [12,13,14,15,18]. It is therefore easy to understand how a mutation in this protein could lead to uncontrolled cell growth and, consequently, the development of new formations. Several mutations can affect Merlin; the most common ones—which are associated with a more severe manifestation of the disease—appear to be nonsense and frameshift mutations, which are also correlated with an earlier age of tumor onset [15,18].

### 5.2. Comparison Between Our Case Report and the Literature Review Data

Our case, compared to the data found following our analysis of the scientific literature, deviates slightly, and this reflects the uniqueness of this case report.

Data from our review of the literature assert that a schwannoma is a rare tumor that develops in young people, with a peak presentation between the ages of 20 and 30, with some authors reporting cases up to 50 years of age, but it is very rare in children and pediatric patients [1,2,4,5,16,20,21,22,23,24,25,26].

Our patient is a 17-year-old boy who reported noticing the onset of the lesion many years earlier, and according to other colleagues who had examined him, it was considered a mucocele or another lesion of traumatic origin, so we can assume that the lesion appeared when he was 10 years old. The age of presentation in our case is not in line with literature review data.

Most schwannomas, due to their clinical presentation and the fact that they often develop following an injury, are mistaken with exophytic traumatic lesions, and in most cases they remain undiagnosed because these lesions, considered benign, often are not biopsied [1,2,3,4,5,6,10,11,16,17,18,19,20,21,22,23,24,25,26]. In our case, a history of trauma with the teeth on the lower lip mucosa was reported by the parents; however, the biopsy overturned the diagnostic hypothesis of a traumatic lesion, as the histology revealed positivity for the S-100 protein, a typical neuronal marker; positivity for this marker is confirmed in 100% of schwannomas [1,2,4,5,6,10,11,16,21,23,26].

Another fact that makes our case particularly unique and interesting is the location, specifically the lower lip: the data found in the literature report that schwannomas mainly occur in the head–neck region, with the most typical form affecting the eighth pair of cranial nerves. Oral location, on the other hand, is extremely rare, occurring in only 1–2% of cases, compared to about 25–48% in the head–neck region [1,2,3,4,5,6,7,10,11,16,17,19,20,21,23,24,25,26]. When the oral cavity is involved, the tongue is the site most hit [1,2,3,4,5,6,10,16,17,21,22,24,25], not the lip as in our case, which is uniformly recognized as the least affected site.

In our literature review, we found about 12 documented cases of schwannoma of the lip all around the world [2,21,22,23,24,25].

Excision for biopsy analysis is the best method for treating the lesion. Since the most common form is encapsulated, excision itself does not present a significant challenge in the hands of an experienced surgeon; the real difficulty of the procedure lies in not cutting the nerve from which the tumor originates. Cutting the nerve can lead to impairments and loss of sensation or motor function in the affected area [1]. However, in the documented cases of lip schwannoma no paresthesia was reported after biopsy, as in our case.

Due to the presence of the capsule, recurrence is also rare, although these data are debated in the literature: we did not find a definitive stance on the recurrence rate, as for many authors, if excision is performed with complete removal of the capsule, it does not lead to new lesions, while for others, the recurrence rate is intrinsic to the tumor itself [1,2,5,6,10,11,16,19,21,22,23,24,25,26]. In our case, the lesion recurred but with much smaller dimensions than the initial size, which was one of the reasons a second incision was not performed, as it was much more manageable.

In the diagnostic process, the use of ultrasound evaluation for oral or lip schwannoma is not reported, so our case is the first in the literature in which this instrumental investigation was used. Ultrasounds provide a clear view of the margins of the lesion, and in our specific case it was used to determine if the lesion itself was surrounded by noble structures—blood vessels and nerves—that could be hurt during the biopsy. As you can see in Figure 2a,b, reported in the Case Presentation section, noble structures were not present around the lesion. Moreover, the second ultrasound examination with color power Doppler investigation (Figure 5) showed no infiltration of vessels and nerves and specific blood circulation around the lesion. This fact contributes to operating in a safe way, lowering the possibility of causing injury to the patient. Even more, ultrasounds are safe and economic, and images are quickly obtained [27].

The last aspect of our analysis is the link between the development of schwannomas, particularly multiple ones, and Neurofibromatosis type 2. This is an autosomal dominant disease with an incidence of 1 in 33,000 inhabitants, characterized by the development of neural tumors, with vestibular schwannoma being the most represented. Although there is full agreement in the literature that schwannomas originate from a double random mutation—of which, as discussed earlier, frameshift mutations are the most common—in 4% of cases these lesions are multiple and associated with NF2 [15].

Our patient presented only one lesion. RMI performed by other physicians did not reveal other SNC suspected neoformations.

This review led us to pay closer attention to our patient to monitor the possible development of a syndromic condition, which, fortunately, has not occurred so far.

We can conclude that, in the literature, due to the very few cases documented on the lips, there is not a diagnostic and therapeutic protocol to follow.

### 5.3. Difference Between Pediatric and Adult Labial Schwannomas

This type of lesion appears to be identical from a clinical and histopathological point of view in both adults and children. The differences between oral schwannoma in pediatric age and in adulthood are essentially due to the following: (1) etiopathogenetic factor: as previously mentioned, trauma to the lip can induce nerve damage, which instead of determining fibroblast proliferation and giving rise to a fibroma or determining a lesion to the salivary glands, leading to the formation of a mucocele, can lead to a hyperproliferation of Schwann cells that surface superficially on the labial and perilabial mucosa region. In fact, children are more prone to labial trauma than adults [20,21,23]; (2) connection with systemic diseases: if this lesion occurs at a young age, it is more likely to be associated with genetic defects (i.e., NF2) and multiple manifestations in sites other than the oral cavity [15,21,23,25].

### 5.4. New Diagnostic Flow Chart Proposal

Given the particularity of the observed case, through our personal experience and our review of the literature, we feel we can propose a diagnostic procedure in case of suspected normochromic exophytic labial lesions for schwannoma:

(1) Objective examination that includes inspection and palpation of the tissues (a fluctuating consistency may point us towards a cystic lesion, while a tense–elastic consistency may point us towards a solid neoformation) and evaluation by autofluorescence to exclude solid malignant or potentially malignant lesions [28]. Dark-green or black color of the illuminated mucosa could be related to malignancies;
(2) Perform an intraoral ultrasound examination with possible eco-Doppler to highlight the intra- and perilesional blood vessels and understand if the content of the lesion is liquid or solid;
(3) Excisional biopsy for histological evaluation;
(4) MRI of the head and neck area to be performed immediately after the possible histological confirmation and every 1–2 years as a follow-up to intercept any other neo-formations or lesions that affect nervous tissues;
(5) Genetic investigation to evaluate any alterations of the nervous tissue and to discover the syndromic picture associated with the oral schwannoma.

**Figure 7:**
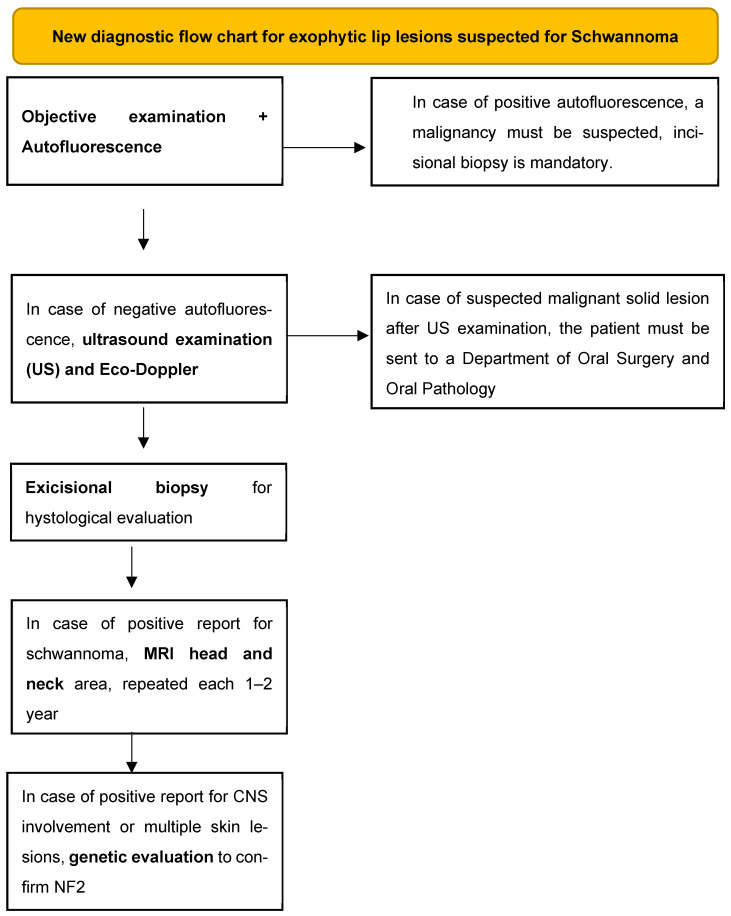
Flow chart that outlines the new diagnostic process proposal explained in the text.

In cases of these last two points (MRI shows lesions on the head–neck district and/or findings of genetic mutations), consider counseling with pediatricians, geneticists, and neurologists (Figure 7).

## 6. Conclusions

The literature review we conducted allowed us to compare our case with the data found in the literature. We believe that our patient’s case is extremely interesting because it is exceedingly rare. Schwannoma is considered to be a tumor typically seen in young adults, with an average age of around 20–30 at the time of diagnosis, while in our case the patient is an adolescent with a long medical history. Furthermore, if the presentation in the oral cavity is already uncommon, its appearance on the lip is established as, in general, the rarest form; we did not find the exact percentage of schwannoma presentation on the lips, but it is quite well accepted by all the authors that the lip is the most uncommon oral site to be localized. The diagnostic delay, which has also been noted in the literature and is due to the nonspecific nature of the lesion, in our case was certainly caused by the fact that no clinician considered it necessary to biopsy and analyze the lesion, allowing it to persist for years with episodes of exacerbation and stabilization, mistaking it for a traumatic lesion (mucocele, mucous extravasation cyst) that commonly occurs in children. Therefore, our work aims to encourage all clinicians not to overlook lesions that may seem trivial at first glance and to provide a practical diagnostic pathway for the evaluation of such lesions.

## Figures and Tables

**Table 1:** Inclusion criteria.

Inclusion Criteria
Case reports, Clinical trials, Meta-analyses, Reviews, Systematic reviews Timeframe: 2005–2024 Free full text English language Human patients

**Table 2:** Summary of the case reports used to conduct the literature review, in order of appearance in the text.

Year	Author	Title	Reference
2009	López-Carriches et al.	Schwannoma located in the palate: Clinical case and literature review	[1]
2009	Martins et al.	Intra-oral schwannoma: Case report and literature review	[2]
2021	Nassehi et al.	Floor of mouth schwannoma mimicking a salivary gland neoplasm: A report of the case and review of the literature	[3]
2017	Salehinejad et al.	Intraoral ancient schwannoma: A systematic review of the case reports	[5]
2023	Zhang et al.	Maxillary gingival neurolemmoma: a case report and literature review	[6]
2008	Baderca et al.	Schwannoma of the lip: Case report and review of the literature	[10]
2019	Dokania et al.	Palatal schwannoma: An analysis of 45 literature reports and of an illustrative case	[11]
2009	Subhashraj et al.	Ancient schwannoma arising from mental nerve. A case report and review.	[17]
2021	Thakur et al.	Lingual schwannoma in a 4-year-old female baby	[20]
2024	Maulana et al.	A rare case of upper lip schwannoma: A case report with analysis of the histological, immunohistochemical and pathogenesis aspects	[21]
2013	Bayindir et al.	Schwannoma with an Uncommon Upper Lip Location and Literature Review	[22]
2016	Hajong et al.	Schwannoma of upper lip: Report of a rare case in a rare age group	[23]
2019	Desai et al.	An unexpected and rare outcome of a common nodular mass on upper lip in a pediatric patient with a history of trauma–Schwannoma	[24]
2020	de Menezes et al.	Schwannoma of the lower lip mimicking a mucocele in children	[25]
2021	Alhammad et al.	Schwannoma of the lower lip mimicking a mucocele in children	[26]

## Data Availability

The original contributions presented in the study are included in the article, further inquiries can be directed to the corresponding author.

## References

[1] López-Carriches C., Baca-Pérez-Bryan R., Montalvo-Montero S. (2009). Schwannoma located in the palate: Clinical case and literature review. Med. Oral Patol. Oral Cir. Bucal.

[2] Martins M.D., Anunciato De Jesus L., Fernandes K.P.S., Bussadori S.K., Taghloubi S.A., Martins M.A.T. (2009). Intra-oral schwannoma: Case report and literature review. Indian J. Dent. Res..

[3] Nassehi Y., Rashid A., Pitiyage G., Jayaram R. (2021). Floor of mouth schwannoma mimicking a salivary gland neoplasm: A report of the case and review of the literature. BMJ Case Rep..

[4] Phulware R.H., Sardana R., Chauhan D.S., Ahuja A., Bhardwaj M. (2022). Extracranial Schwannomas of the Head and Neck: A Literature Review and Audit of Diagnosed Cases Over a Period of Eight Years. Head Neck Pathol..

[5] Salehinejad J., Sahabnasagh Z., Saghafi S., Sahebnasagh Z., Amiri N. (2017). Intraoral ancient schwannoma: A systematic review of the case reports. Dent. Res. J..

[6] Zhang X., Gao Q., Xuan Y. (2023). Maxillary gingival neurolemmoma: A case report and literature review. BMC Oral Health.

[7] Cortes-Santiago N., Patel K. (2021). Review of Pediatric Head and Neck Neoplasms that Raise the Possibility of a Cancer Predisposition Syndrome. Head Neck Pathol..

[8] Ruggieri M., Praticò A.D., Serra A., Maiolino L., Cocuzza S., Di Mauro P., Licciardello L., Milone P., Privitera G., Belfiore G. (2016). Childhood neurofibromatosis type 2 (NF2) and related disorders: From bench to bedside and biologically targeted therapies. Acta Otorhinolaryngol. Ital..

[9] Bachir S., Shah S., Shapiro S., Koehler A., Mahammedi A., Samy R.N., Zuccarello M., Schorry E., Sengupta S. (2021). Neurofibromatosis type 2 (NF2) and the implications for vestibular schwannoma and meningioma pathogenesis. Int. J. Mol. Sci..

[10] Baderca F., Cojocaru S., Lazǎr E., Lǎzureanu C., Faur A., Lighezan R., Alexa A., Raica M., Vǎlean M., Balica N. (2008). Schwannoma of the lip: Case report and review of the literature. Rom. J. Morphol. Embryol..

[11] Dokania V., Rajguru A., Mayashankar V., Mukherjee I., Jaipuria B., Shere D. (2019). Palatal schwannoma: An analysis of 45 literature reports and of an illustrative case. Int. Arch. Otorhinolaryngol..

[12] Helbing D.L., Schulz A., Morrison H. (2020). Pathomechanisms in schwannoma development and progression. Oncogene.

[13] Karajannis M.A., Ferner R.E. (2015). Neurofibromatosis-related tumors: Emerging biology and therapies. Curr. Opin. Pediatr..

[14] Kim B.H., Chung Y.H., Woo T.G., Kang S.M., Park S., Kim M., Park B.J. (2024). NF2-Related Schwannomatosis (NF2): Molecular Insights and Therapeutic Avenues. Int. J. Mol. Sci..

[15] Pećina-Šlaus N. (2013). Merlin, the NF2 gene product. Pathol. Oncol. Res..

[16] Sitenga J.L., Aird G.A., Nguyen A., Vaudreuil A., Huerter C. (2017). Clinical features and surgical treatment of schwannoma affecting the base of the tongue: A systematic review. Int. Arch. Otorhinolaryngol..

[17] Subhashraj K., Balanand S., Pajaniammalle S. (2009). Ancient schwannoma arising from mental nerve. A case report and review. Med. Oral Patol. Oral Cir. Bucal.

[18] Tamura R. (2021). Current understanding of neurofibromatosis type 1, 2, and schwannomatosis. Int. J. Mol. Sci..

[19] Sitenga J., Aird G., Vaudreuil A., Huerter C.J. (2018). Clinical features and management of schwannoma affecting the upper and lower lips. Int. J. Dermatol..

[20] Thakur V.K., Rahul S.K., Kumar B., Hasan Z., Yadav R., Chaubey D., Prasad R., Keshri R. (2021). Lingual schwannoma in a 4-year-old female baby. J. Indira Gandhi Inst. Med. Sci..

[21] Maulana R., Pahlevi M.R., Rosanto Y.B., Sejati B.P., Hasan C.Y. (2024). A rare case of upper lip schwannoma: A case report with analysis of the histological, immunohistochemical and pathogenesis aspects. Int. J. Surg. Case Rep..

[22] Bayindir T., Kalcioglu M.T., Cicek M.T., Karadag N., Karaman A. (2013). Schwannoma with an Uncommon Upper Lip Location and Literature Review. Case Rep. Otolaryngol..

[23] Hajong R., Hajong D., Naku N., Sharma G., Boruah M. (2016). Schwannoma of upper lip: Report of a rare case in a rare age group. J. Clin. Diagnostic Res..

[24] Desai J. (2019). An unexpected and rare outcome of a common nodular mass on upper lip in a pediatric patient with a history of trauma–Schwannoma. Natl. J. Maxillofac. Surg..

[25] de Menezes B.N.F., Cunha J.L.S., Chaves-Júnior S.d.C., Bezerra B.T. (2020). Schwannoma of the lower lip mimicking a mucocele in children. Autops. Case Reports.

[26] Alhammad G., Alsaad A., Aljohani T., Alajlan A. (2021). Schwannoma of the Lower Lip: A Case Report of an Unusual Presentation. Case Rep. Dermatol..

[27] Wu W.T., Chang K.V., Hsu Y.C., Yang Y.C., Hsu P.C. (2020). Ultrasound Imaging for a Rare Cause of Sciatica: A Schwannoma of the Sciatic Nerve. Cureus.

[28] Lau J., O G., Warnakulasuriya S., Balasubramaniam R., Frydrych A., Kujan O. (2024). Adjunctive aids for the detection of oral squamous cell carcinoma and oral potentially malignant disorders: A systematic review of systematic reviews. Jpn. Dent. Sci. Rev..

